# Epidemiological characteristics of human prion diseases

**DOI:** 10.1186/s40249-016-0143-8

**Published:** 2016-06-02

**Authors:** Cao Chen, Xiao-Ping Dong

**Affiliations:** State Key Laboratory for Infectious Disease Prevention and Control, National Institute for Viral Disease Control and Prevention, Chinese Center for Disease Control and Prevention, Changbai Rd 155, Beijing, 102206 China; Collaborative Innovation Center for Diagnosis and Treatment of Infectious Diseases, Zhejiang University, Hangzhou, 310003 China; Chinese Academy of Sciences Key Laboratory of Pathogenic Microbiology and Immunology, Institute of Microbiology, Chinese Academy of Sciences, Beijing, 100101 China

**Keywords:** Prion disease, Epidemiology, Creutzfeldt-Jakob disease, Surveillance

## Abstract

Human prion diseases are a group of transmissible, progressive, and invariably fatal neurodegenerative disorders, which include Kuru, Creutzfeldt-Jakob disease (CJD), Gerstmann-Sträussler-Scheinker syndrome, and fatal familial insomnia. Human prion diseases affect approximately 1–2 persons per million worldwide annually, occurring in sporadic, inherited, and acquired forms. These diseases have attracted both scientific and public attention not only because of their mysterious pathogen, but also due to their considerable threat to public health since the emergence of the variant CJD.

There are still no specific therapeutic and prophylactic interventions available for prion diseases, thus active surveillance of human prion diseases is critical for disease control and prevention. Since 1993, CJD surveillance systems have been established in many countries and regions, and several long-term multinational cooperative projects have been conducted.

In this paper, the epidemiological characteristics of various human prion diseases and the active surveillance systems pertaining to them in different countries and regions are summarized and reviewed.

**Electronic supplementary material:**

The online version of this article (doi:10.1186/s40249-016-0143-8) contains supplementary material, which is available to authorized users.

## Multilingual abstract

Please see Additional file 1 for translations of the abstract into the six official working languages of the United Nations.

## Background

Human prion diseases, also named transmissible spongiform encephalopathies (TSEs), are fatal neurodegenerative disorders, which include Kuru, Creutzfeldt-Jakob disease (CJD), Gerstmann-Sträussler-Scheinker syndrome (GSS), and fatal familial insomnia (FFI). These various subtypes have different characteristics based on the onset times/durations of the disease, genetic/family histories of the patients, clinical manifestations, neuropathology, and scrapie-like prion protein (PrP^Sc^) molecular features [1, 2]. It is widely accepted that TSEs result from the conformational conversion of a normal cellular prion protein (PrP^C^) into an abnormal misfolded pathological form (PrP^Sc^). An accumulation of PrP^Sc^ leads to the onset of TSEs, which attack the central nervous system, resulting in progressive neuronal degeneration and neuronal vacuolation [3].

Currently, Kuru is virtually extinct due to a ban on ritualistic cannibalism in the area of Papua New Guinea where it was endemic. Most of the human prion diseases are CJD, which consist of three main catalogues: sporadic, genetic, and acquired [1]. Approximately 85–90 % of CJD cases occur sporadically and affect 1–1.5 people per million annually [4]. Familial/genetic CJD (fCJD/gCJD) account for about 10 % of CJD cases worldwide [5]. Acquired prion diseases include variant CJD (vCJD) and iatrogenic CJD (iCJD), and are observed in 2–5 % of CJD cases. Depending on the origin of the causative agent, human prion diseases can be divided into two groups: caused by prions originating internally, such as in the case of fCJD/gCJD, GSS, and FFI; or infected by external prions, such as in the case of Kuru, iCJD, and vCJD [6].

The pathogenesis of sporadic CJD (sCJD) is little known. Many case–control studies on the risk factors for sCJD have been conducted, with varying results, but no consistent data are available [7–12]. However, the opportunity of external prion infection is still not easy to be excluded definitely during long life-span [13].

As there are no specific therapeutic and prophylactic interventions available for prion diseases, active surveillance is critical for the control and prevention of human prion diseases, especially those diseases caused by animal-derived prion agents. Since 1993, many national CJD surveillance systems have been established and several multinational cooperative organizations have also been set up for CJD surveillance and research [14–18]. In the present review, the epidemiological characteristics of various subtypes of human prion diseases and the active surveillance systems pertaining to them are summarized and discussed.

## Epidemiological characteristics of human prion diseases

### sCJD

CJD was first described in the early 1920s [19, 20]. The predominant subtype of human prion diseases, sCJD, occurs equally in both sexes with a peak age of onset between 60 and 69 years [21–23]. sCJD occurs all year round, with no seasonal specificity. Typical clinical symptoms include progressive dementia, accompanied by visual and cerebellum function abnormalities, myoclonia, pyramidal and extrapyramidal dysfunction, or akinetic mutism [2, 21]. The duration of sCJD cases is relatively short. The median survival time of Chinese sCJD cases is 7.1 months (range: 1.0–23.3) and 78.5 % of patients die within one year of onset [24]. These data are comparable with that of Western countries but differ to data from Japan. A study conducted by the European CJD Surveillance Network (EuroCJD) involving 2,451 sCJD patients, who died between 31 December 1992 and 31 December 2002, revealed that the median survival time was five months (range: 1–81) and that 85.8 % of patients died within one year of onset [25]. In Argentina, the median disease duration of sCJD (calculated using 150 definite and probable cases from available data) is 4.6 months (range: 1–70) [17]. On the contrary, a survey by the Japanese CJD surveillance program showed significantly longer disease durations in Japanese patients with prion diseases (most of them with the sCJD subtype), in which the mean disease duration of 855 patients was 17.4 months and only 46.0 % died within one year of onset. This is likely attributed to the country’s healthcare system, which provides intensive life-sustaining treatment for patients [26]. There is no accessible data for sCJD cases in South Asia or Africa.

**Fig. 1:**
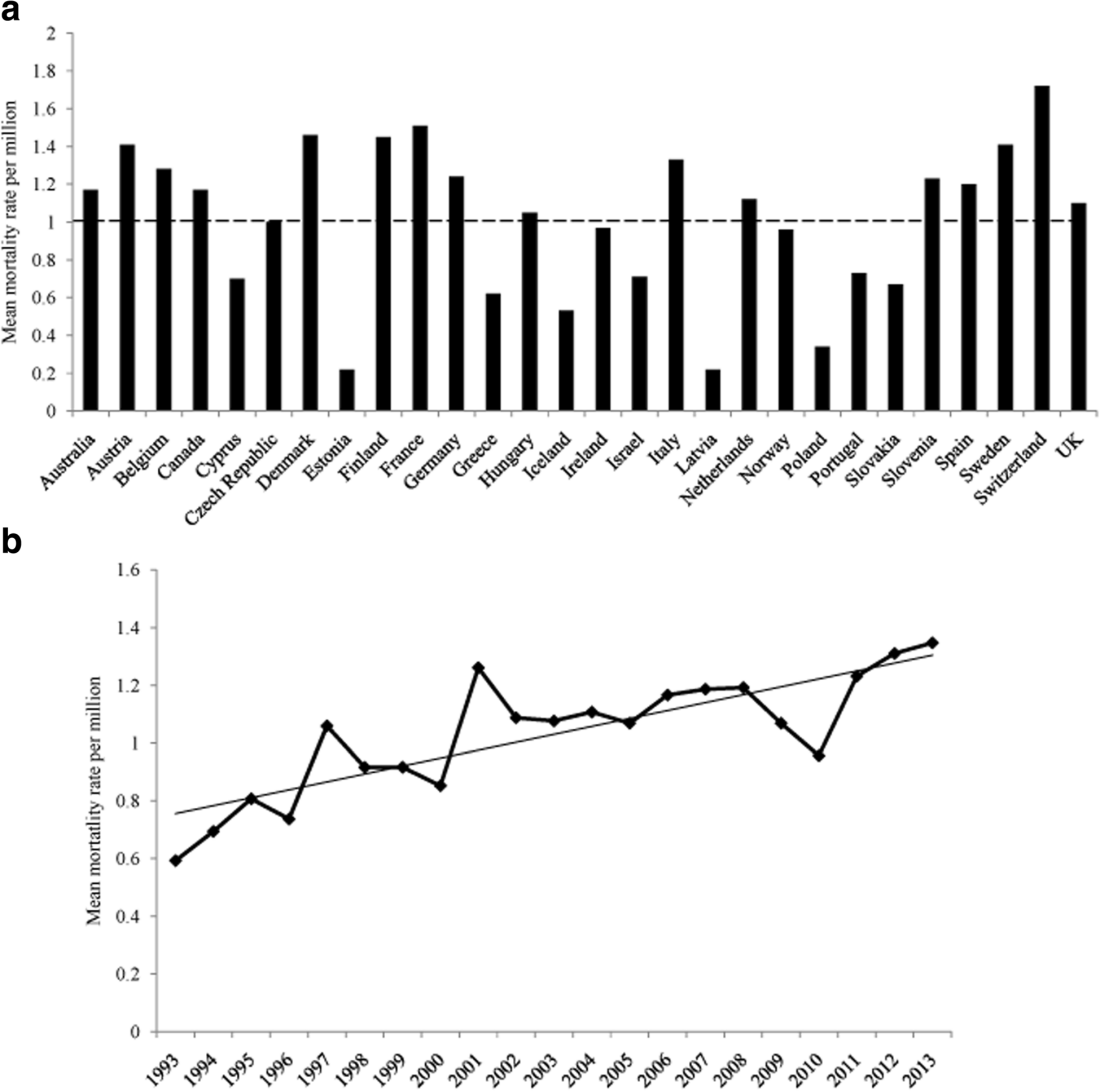
The mean mortality rates per million from sCJD according to the data of the CJD International Surveillance Network from 1993 to 2013. **a** Data from the member states of the CJD International Surveillance Network. The mortality rate of one per million is marked with a dotted line. **b** The mean mortality rates per million of all member states. Solid line represents the fitting trend

An international study on the epidemiologic characteristics of sCJD involving 3,720 sCJD cases from nine European countries, as well as from Australia and Canada, revealed that the overall annual mortality rate from sCJD is 1.39 per million [27]. In Japan, the age-adjusted mortality rates have increased from 1979 to 2004, with the annual mortality rate of 1.48 per million in 2004 [28]. The Chinese CJD surveillance network reported that the annual CJD morbidity rate in Beijing is 0.91 per million [29]. The recognition of CJD clinically and the undertaking of national CJD surveillance influences a country’s mortality rate. According to the latest data from the CJD International Surveillance Network (formerly the EuroCJD), the countries with the highest mean mortality rates per million from sCJD are France and Switzerland (1.51 and 1.72, respectively). The mean mortality rates per million from sCJD from 1993 to 2013 of some countries are shown in Fig. 1a; 16 out of 28 countries have a mean mortality rate per million greater than 1.0. Averaging the annual data from all countries in the CJD International Surveillance Network from 1993 to 2013 results in the mortality rates per million increased (see Fig. 1b). In the United States (US), the annual mortality rate is approximately one per million based on data from 1979 to 2006 [30]. However, it is important to note that most CJD cases from these data were European Americans (94.6 %) and that the age-adjusted incidence of whites is 2.7 times higher than that of African Americans (1.04 and 0.40, respectively) [30], and is also higher than that of Native Americans and Alaska Natives (0.47) [31]. Although reasons for such disparities are unclear, it is possible that genetic differences and/or under diagnosis among non-white patients are two associated factors.

### Genetic human prion diseases

The human gene encoding for the prion protein (PrP), *PRNP*, is located on chromosome 20p12 in humans. To date, more than 40 mutations in the *PRNP* gene have been directly linked to familial prion diseases, including fCJD/gCJD, GSS, and FFI [32]. These mutations include point mutations in the *PRNP* sequence, and octapeptide repeat insertions or deletions in PrP’s N-terminus. Several epidemiological surveys report that some patients with genetic prion diseases lack definite family history [33, 34].

The distribution and frequency of mutations in the *PRNP* gene vary significantly among geographical areas and human races. In gCJD/fCJD, the three most common *PRNP* mutations in European Caucasians, North Americans, and Australians are E200K, V210I, and D178N/129 V [34]. Interestingly, E200K is the only mutation in the *PRNP* gene reported in the Slovak population, whereas only one E200K gCJD case was identified in the population of Switzerland between 1996 and 2002, and no E200K gCJD has been reported after this. The proportionate incidence of the V210I mutation in the Italian population is obviously higher than that in other ten countries (50 out of 69 V210I gCJD cases are observed in Italy), according to data from the EuroCJD 1993–2002 [35]. In Japan, the most frequently observed mutation in the *PRNP* gene is V180I, followed by E200K and M232R [16], while the mutations T188K and E200K are most frequent in China [36]. Five mutations in codon 180, three in codon 200, two in codon 203, and two in codon 232 have been identified in the Republic of Korea [37–42]. The frequency of gCJD cases in the Republic of Korea is similar to that in Japan, but differs from that in China (see Table 1) [43]. In addition, the V210I mutation is prevalent in European countries but is rare in East Asian populations. M232R, which is one of the most frequently described gCJD-associated mutations in the Japanese, is rarely identified among Europeans.

**Table 1:** The distribution of genetic prion diseases in European and East Asians countries

Diseases	Mutations	Europeans [27]	East Asians [10, 29, 31–35]				*P-*value^a^
		Total (*n* = 420)	China (*n =* 62)	Japan (*n =* 216)	Korea (*n* = 15)	Total (*n* = 293)	
gCJD	Insertion	39	2	3	0	5	P < 0.001
	N171S-129 V	1	0	0	0	0	n.s.
	D178N-129 V	16	0	1	0	1	*P* < 0.01
	V180I-129 M	1	1	89	5	95	*P* < 0.001
	T188A-129 M	3	0	0	0	0	n.s.
	T188K-129 M	0	16	0	0	16	*P* < 0.001
	E196K-129 M/V	5	1	0	0	1	n.s.
	E200K-129 M/V	175	9	37	3	49	*P* < 0.001
	V203I-129 M	5	1	2	2	5	n.s.
	R208H-129 M	2	2	1	0	3	n.s.
	V210I-129 M	69	0	0	0	0	*P* < 0.001
	E211Q-129 M	4	0	0	0	0	n.s.
	M232R-129 M	0	0	33	2	35	*P* < 0.001
GSS	P102L-129 M	24	3	39	2	44	*P* < 0.001
	P105L-129 M	0	0	5	0	5	*P* < 0.05
	A117V-129 V	12	0	0	0	0	*P* < 0.01
FFI	D178N-129 M	64	27	3	1	31	n.s.

^a^The differences of frequencies of PRNP mutations between Europeans and East Asians were measured by the Chi-square test or Fisher’s exact test. gCJD, genetic Creutzfeldz-Jakob diseases; GSS, Gerstmann-Sträussler-Scheinker syndrome; FFI, fatal familial insomnia; n.s., not significant

The distribution and frequency of mutations in the *PRNP* gene causing GSS are also clearly distinct between Caucasians and East Asians. Although the most common mutation causing GSS in Caucasian and East Asian patients is P102L, the mutation P105L is only observed in East Asian populations, especially in the Japanese, while the mutation A117V is exclusively reported in Caucasians.

FFIs caused by the mutation D178N in the *PRNP* gene associated with the M129 genotype have been reported worldwide. However, there are also clear geographical and race-associated variations. FFI is predominant in some regions of Europe, such as in Spain and Germany, where 56.8 % (25/44) and 25 % (17/68) of genetic prion diseases are FFI, respectively [34]. It is worth emphasizing that FFI cases are common in the Han Chinese population, which reaches to the first most common *PRNP* mutation in all identified mutations associated with genetic prion diseases in China [36], revealing a distinct profile compared with those in Japan and Korea (see Table 1). A study further addressing *PRNP* mutations among different ethnic groups is warranted.

The age at onset of genetic prion diseases is often earlier than that of sCJD, ranging from 30 to 55 years for gCJD, 40 to 60 years for GSS, and 20 to 72 years for FFI [43]. Although gCJD cases with point mutations have an earlier median age of death compared with that of sCJD cases, there is no difference between gCJD cases with point mutations and sCJD in the mean duration of the disease [44–46]. Meanwhile, gCJD cases with extra insertional octarepeat sequences, as well as GSS and FFI cases often have a relatively protracted duration of illness in Caucasians [34, 47, 48]. Relatively long clinical durations are also observed in Chinese FFI cases [49] and Japanese GSS patients [26].

In addition to these disease-related mutations, polymorphisms have also been described in PrP [32, 50]. In particular, single nucleotide polymorphisms (SNPs) at codons 129 and 219 of the *PRNP* gene represent susceptibility factors for human prion diseases [51, 52]. The pattern of SNP at codon 129 greatly varies between Caucasians and East Asians. An overwhelming percentage of East Asians (92 % to 94 %) exhibit methionine/methionine homozygote at codon 129 (M129M), but a much lower percentage of Caucasians have this polymorphism (32 % to 45 %) [43]. Homozygosity at codon129 (M/V polymorphism) is a strong risk factor for the development of sCJD in Caucasians [15, 53–55]. Moreover, all vCJD cases with clinical symptoms and genetic analysis worldwide are M129M homozygous [56]. Homozygosity at a different *PRNP* polymorphism, E219K, seems to also be a risk factor for the development of sCJD in Korean and Japanese populations, but not in Caucasian populations [16, 52, 57–59]. In addition, *PRNP* codon 129 polymorphism has obvious effects on the clinical, neuropathological, and pathogenic features of prion disease. For instance, in the population with the D178N mutation in the *PRNP* gene, the codon 129 polymorphism determines the type of disease: people with the M129M mutation suffer from FFI and those with the M129V mutation acquire gCJD [60, 61].

### iCJD

**Fig. 2:**
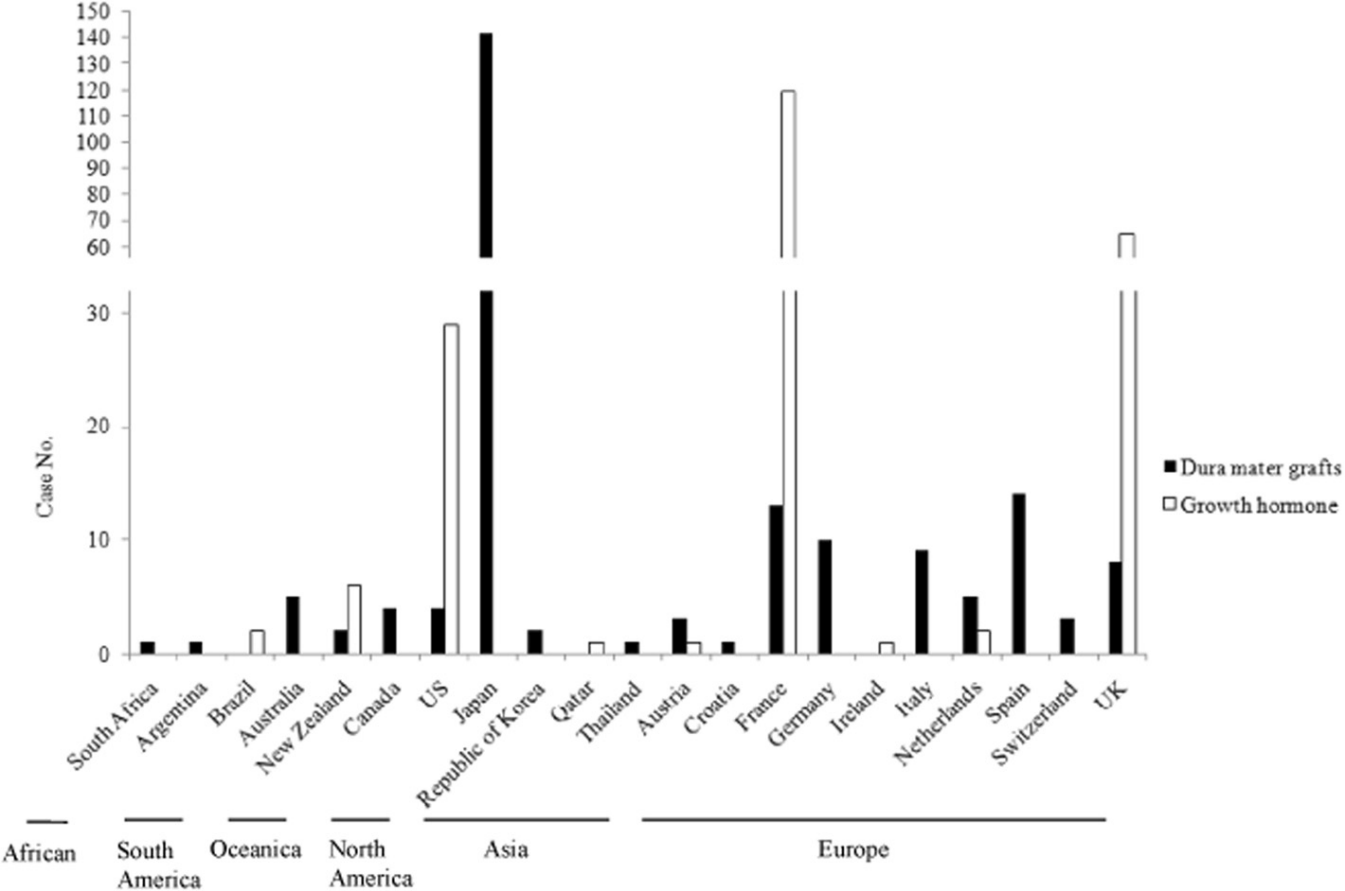
The distribution ofdura mater graft-associated and hGH-related iCJD casesglobally

In 1974, iatrogenic CJD (iCJD) was firstly described in a person who received cadaveric corneal transplant from a patient with CJD [62]. Since then, several cases of human prion disease have been confirmed to be associated with iatrogenic transmission of CJD by the use of stereotactic intracerebral electroencephalogram needles or neurosurgical instruments [63–67]. Additionally, corneal grafts and the gonadotropin hormone [68, 69] can also cause iCJD. Because of the long incubation time of iCJD, it is usually very difficult to attribute the disease to a special medical service in the lifespan of a patient. Therefore, the numbers of iCJD cases might be underestimated. Historically, large outbreaks of human iCJD cases have been reported via two different medical pathways: one is cadavericdura mater grafts [16], and the other is intramuscular injection of contaminated cadaveric pituitary-derived human growth hormone (hGH) and gonadotropin hormone [70]. Since the first identification of dura mater graft-associated iCJD in 1987, at least 228 cases have been reported worldwide. Nearly two thirds of cases come from Japan, with some European countries, such as France, Spain, Germany, Italy, and the Netherlands, also reporting iCJD. Cases have also been reported from Australia (five cases), South Africa (one case), Argentina (one case), the US (four cases), and South Korea (two cases) [68]. At least 226 hGH-related iCJD caseshave been reported worldwide, mostly in France (119 cases), the United Kingdom, UK (65 cases) and the US (29 cases), and a few cases in Brazil (two cases), New Zealand (six cases) and Qatar (one case) [68]. The global distribution of iCJD cases associated with dura mater grafts and the hGH is shown in Fig. 2. With the availability of recombinant hGH and the initiation of separated processing of individual dura mater grafts, the transmission pathways of these two kinds of iCJD have been successfully eradicated and almost no new cases have been reported in the past few years [70]. So far, there have been no reports of dura mater graft-associated or hGH-related iCJD in China.

The clinicopathological features of dura mater graft-associated iCJD cases resemble those of sCJD cases. However, in Japan, approximately one third of these cases have atypical features (slow progression, non-characteristic electroencephalogram tracings, plaque deposition, and an atypical prion protein molecular signature on Western blots), suggesting the possibility of two different types of infectious agents [71, 72]. The incubation periods are in the range of 1.3 to 30 years (mean: 12) worldwide [68]. The clinicopathological features of hGH-related iCJD cases resemble those of Kuru. The incubation periods vary from five to 42 years worldwide (mean:17) [68]. Those with the M129M mutation are at risk for acquiring hGH-related iCJD in France and in the US, but not in the UK [68, 73, 74].

### vCJD

**Fig. 3:**
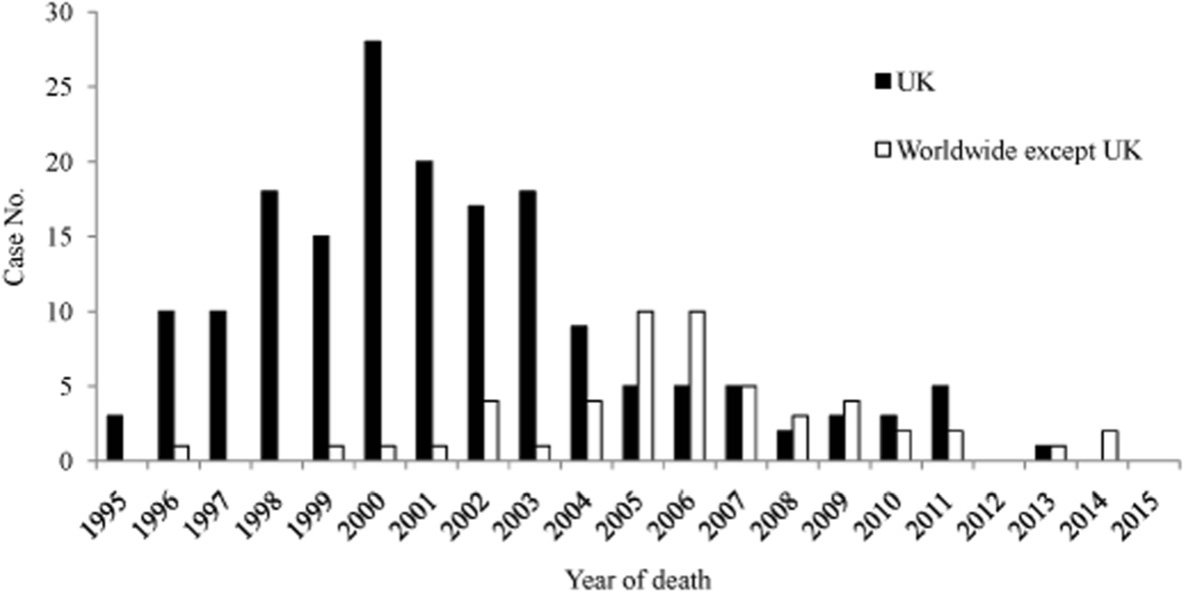
Number of deaths from vCJD in the UK and outside of the UK from 1995 to 2015

The first 10 vCJD cases were reported in April 1996 in the UK [70, 75]. As of April 2015, 229 vCJD cases have been reported from seven European (UK, France, Spain, Republic of Ireland, Netherlands, Italy, and Portugal) and five non-European countries or regions (US, Canada, Saudi Arabia, Japan, and China-Taiwan). Among them, 177 cases were reported from the UK [76]. In 2000, the annual number of deaths from vCJD in the UK reached a peak of 28. Since 2006, the annual deaths from vCJD have dramatically reduced, with 2–5 from 2006 to 2011, none in 2012, and only one in 2013 [77]. Since 2014, no more vCJD cases have been reported (see Fig. 3). Outside of the UK, France is the most affected country, with 27 vCJD cases reported from 1996 to 2014, which is thought to be related to the peak in the volume of beef imports from the UK from 1985 to 1995 [78]. This potential relationship is shown by the peak of the number of deaths from vCJD in France in 2005, five years after a similar peak in the number of deaths occurred in the UK [78, 79]. Additionally, three East Asian vCJD cases has been identified in Hong Kong SAR [80], Japan [81, 82], and China-Taiwan [83]. All three are assumed to be imported cases from the UK due to patients who either previously resided in or travelled to the UK bringing them in. The median age of onset is 27 years (range: 12–74) and the median duration of the disease is 14 months in the UK (range: 6–40). In France, despite a median age of onset is 35 years (range: 18–57), which is higher than in the UK, all other data are similar [84]. The disease duration in Asians (mean 28.3, range 14–43 months) seems to be longer than in patients in the UK and France [83]. A further study supports the hypothesis that a single strain of infectious agent is responsible for all vCJD infections [85]. Probable secondary transmission of vCJD via blood transfusions has been reported [86]. Animal experiments have shown that the M129V heterozygote is less sensitive to the transmission of vCJD and bovine spongiform encephalopathy (BSE) agents [87, 88]. Thus, vCJD with long incubation periods in individuals with M129V and V129Vgenotypes and secondary iatrogenic transmission of vCJD are still serious public health concerns [89–93].

## Surveillance of human prion diseases

**Fig. 4:**
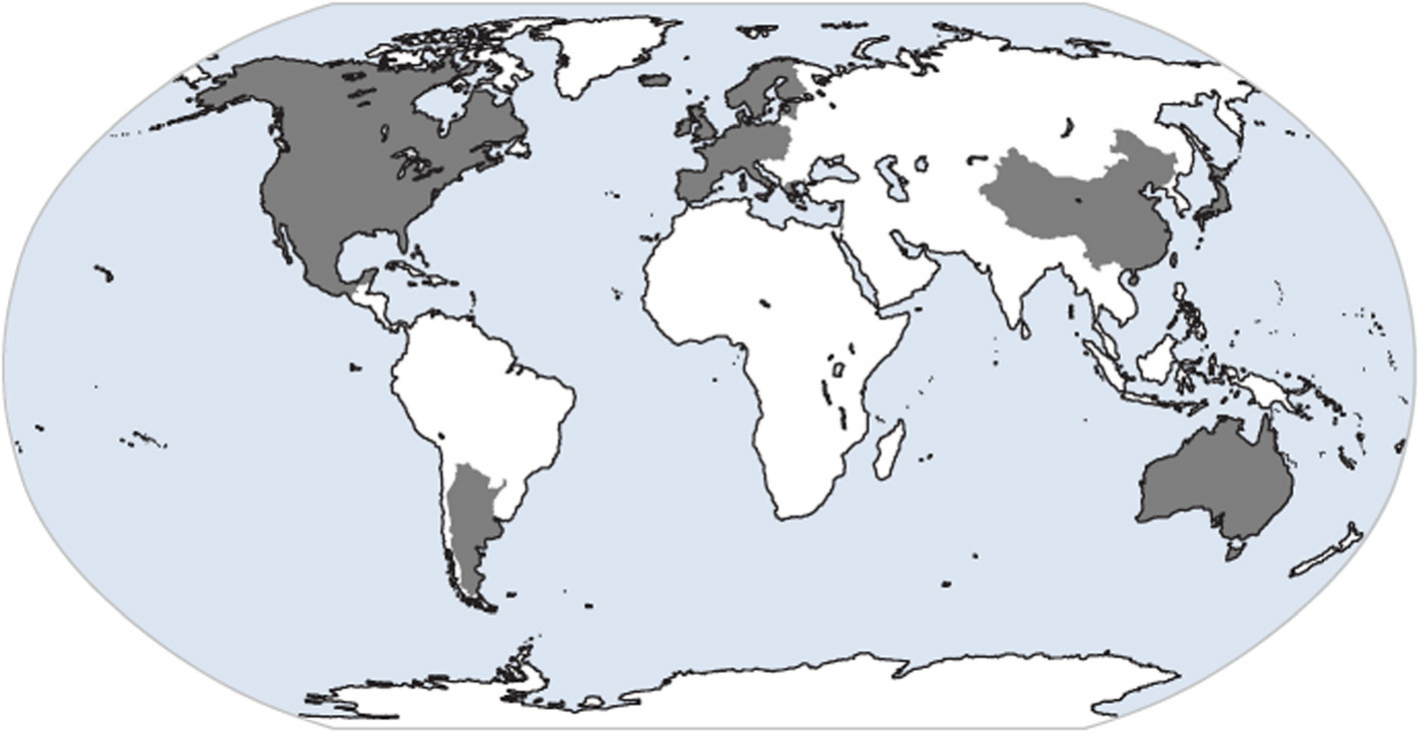
Countries (marked in gray) where active CJD surveillance is officially conducted under the framework of the CJD International Surveillance Network

Due to the impact that the BSE outbreak and the emergence of vCJD has had on public health, many countries and regions have initiated or re-initiated their surveillance programs for human prion diseases. Initially, two major surveillance networks for human prion diseases were created by the European Commission. One is the EuroCJD established in 1993 by seven countries (Austria, France, Germany, Italy, Netherlands, Slovakia, and the UK), which was later expanded to other European and non-European countries such as Australia, Canada, and Spain. The other one is NeuroCJD initiated in 1998, which includes all the other European countries and Israel [18, 34, 79]. In 2008, the CJD International Surveillance Network (formerly EuroCJD) was launched and funded by the European Center for Disease Prevention and Control (CDC) [94, 95]. The network includes 28 collaborating centers from European Union (EU) Member States, European Free Trade Association countries, and eight non-EU countries/regions (Argentina, Australia, Japan, Canada, Mexico, China, Israel, USA, and China-Taiwan) (see Fig. 4). The primary objective of the network is to identify all cases of vCJD in the EU and provide accurate data on the worldwide incidence of vCJD through collaborations with other non-EU countries.

Besides these major CJD surveillance networks, there are several other CJD surveillance and/or research groups intra- and internationally, including CJD surveillance in Central and Eastern European countries [96]. NeuroPrion, which aims to structure and integrate the efforts of the main European prion research teams for the effective management of prion diseases is funded by the European Commission and has been operating since 2003 [18]. In 1997, the National Prion Disease Pathology Surveillance Center of the US was established at the Case Western Reserve University [97] and the following year, the Canadian CJD surveillance system was initiated by the Public Health Agency of Canada [98]. In 1999, the CJD Surveillance Committee was established at the Kanazawa University of Japan, which started carrying out surveillance of CJD nationwide [16]. The CJD surveillance program in the Republic of Korea was established in 2001 and it is supported by the Korean CDCs [18]. In 2006, the China CJD surveillance program was initiated, which is supported by the Chinese CDC [22].

CJD surveillance systems have some unique features compared with other public health surveillance systems. One is that due to a lack of approved biomarkers for CJDs, specific types of clinical and laboratory approaches are critical to effectively diagnose and monitor CJDs. The other is that CJD surveillance systems are primarily dependent on reports from physicians, especially neurologists and neuropathologists in regional hospitals and medical centers where first visit for most patients. Thus, the experiences of these specialists directly determine the quality of the collected specimens, such as accurate identify the clinical manifestations of CJD or supply the appropriate specimens to CJD surveillance center. Both aspects seriously impact the sensitivity of CJD surveillance.

## Conclusion

Human prion diseases are invariably fatal neurodegenerative disorders. The emergence of the novel prion strain, which include the causative agent of vCJD, has created an important public health concern. New prion strains continually emerge in livestock, and their threats to other domestic animals and humans are uncertain and need long-term evaluation and assessment. Strategies for early diagnosis of and therapies to treat human prion diseases remain unavailable. Therefore, except for developing treatment for prion diseases, the most feasible method to prevent these diseases from spreading, either via human-to-human or zoonotic transmission, is active surveillance and improving the sensitivity and specificity of laboratory diagnostic procedures. This will require more clinicians and experts participating in regional, national, and global systems by investing more of an effort toward reliable and accurate diagnostic methods for the control of prion diseases.

## Additional file

Additional file 1: Multilingual abstracts in the six official working languages of the United Nations. (PDF 201 kb)

## Abbreviations

BSE: bovine spongiform encephalopathy
CDC: center for disease control and prevention
CJD: Creutzfeldt-Jakob disease
EU: European union
EuroCJD: European Creutzfeldt-Jakob disease surveillance network
fCJD: familial Creutzfeldt-Jakob disease
FFI: fatal familial insomnia
gCJD: genetic Creutzfeldt-Jakob disease
GSS: gerstmann-sträussler-scheinker syndrome
hGH: human growthhormone
iCJD: iatrogenic Creutzfeldt-Jakob disease
M129M: methionine/methionine homozygote at codon 129
PRNP: human gene encoding for the major prion protein
PrP: prion protein
PrP^C^: cellular prion protein
PrP^Sc^: scrapie-like prion protein
sCJD: sporadic Creutzfeldt-Jakob disease
SNP: single nucleotide polymorphism
TSEs: transmissible spongiform encephalopathies
UK: United Kingdom
US: United States
vCJD: variant Creutzfeldt-Jakob disease
