# Supplementary material for: Epidemiological characteristics of human prion diseases
Source: Infect Dis Poverty. 2016 Jun 2;5:47. doi: 10.1186/s40249-016-0143-8 (PMC4890484; doi:10.1186/s40249-016-0143-8)
Supplement: Additional file 1: — Multilingual abstracts in the six official working languages of the United Nations. (PDF 201 kb) [file 40249_2016_143_MOESM1_ESM.pdf]

Translation of the abstract into the six official working languages of the United Nations

الخصائص الوبائيّة لأمراض بريون

تساو تشن، شياو بينغ دونغ

ملخّص

أمراض بريون التي تصيب الإنسان هي عبارة عن اضطرابات عصبيّة مُعدية وتتطوّر مع الوقت ودائمة وتشمل مرض كورو (Kuru) وكروتزفيلد جاكوب (CJD) وتناذر غيرستمان-ستراوسلر والأرق الوراثي المميت. تُصيب أمراض بريون ما يقارب شخص أو شخصين لكلّ مليون شخص في جميع أنحاء العالم سنويّا. ويظهر بصورة عشوائيّة ومورّثة ويأخذ عدّة أشكال. حظيت هذه الأمراض باهتمام العلماء وعامة النّاس ليس بسبب العوامل المسببّة الغامضة فقط ولكن بسبب التهديد الكبير الذي تشكله على الصحة منذ ظهور أشكال متعددة من مرض كروتزفيلد جاكوب.

لا تزال هناك تدخلات علاجيّة ووقائيّة متاحة لمعالجة مرض بريون، وهكذا تُعتبر المراقبة النّشطة للأمراض أمرا بالغ الأهميّة للسيطرة عليها والوقاية منها. منذ عام 1993، تمّ وضع أنظمة مراقبة لمرض كروتزفيلد جاكوب في العديد من البلدان والمناطق، كما تم إجراء عدة مشاريع تعاونيّة متعدّدة الجنسيات طويلة المدى.

في هذه الدراسة، تمّ تلخيص الخصائص الوبائيّة لمختلف أمراض بريون وأنظمة المراقبة النّشطة في مختلف البلدان والمناطق إضافة إلى مراجعتها.

Translated from English version into Arabic by Zeineb Trabelsi, through

TRANSLATORS WITHOUT BORDERS

人类朊病毒病的流行病学特点

陈操，董小平

摘要

人类朊病毒病是一类传播性、进行性及不可逆的致死性神经退行性疾病，包括库鲁病、克雅氏病、吉斯特曼斯特劳斯综合征及家族致死性失眠症。全球范围内，人类朊病毒病的发病率为百万分之一到二，包括散发型、遗传型和感染型。这类疾病不仅由于其病原的未知性，更是由于变异型克雅氏病出现带来的公共卫生恐慌而得到科学界及社会的关注。

到目前为止，还没有有效的预防和治疗朊病毒病的方法。因此，主动监测人类朊病毒病是控制和阻止该病的主要手段。从1993年起，许多国家和地区都建立了朊病毒病监测系统，同时，一些跨国的朊病毒病监测和研究的长期合作项目也陆续开展。

本文总结了各种人类朊病毒病的流行病学特点，并对各个国家和地区的主动监测系统进行了综述。

Translated from English version into Chinese by Chen Cao

Caractéristiques épidémiologiques des maladies à prion humaines

Cao Chen, Xiao-Ping Dong

Résumé

Les maladies à prion humaines forment un groupe de maladies neurodégénératives transmissibles, évolutives et invariablement fatales, qui incluent le *kuru*, la maladie de Creutzfeldt-Jakob, le syndrome de Gerstmann-Sträussler-Scheinker et l'insomnie fatale familiale. Ces maladies affectent environ 1 ou 2 personnes sur un million par an dans le monde et se présentent sous des formes sporadiques, héréditaires ou acquises. Elles ont attiré l'attention du public autant que celle des scientifiques à cause de leur mystérieux agent pathogène, mais aussi à cause des menaces considérables qu'elles font peser sur la santé publique depuis l'apparition de la nouvelle

variante de la maladie de Creutzfeldt-Jakob (mvMCJ).
Il n'existe encore aucune intervention thérapeutique ou prophylactique contre les maladies à prions et leur surveillance active est donc indispensable à leur contrôle et à leur prévention. Depuis 1993, des systèmes de surveillance de la maladie de Creutzfeldt-Jakob se sont mis en place dans de nombreux pays et régions et plusieurs projets de coopération internationale ont été menés.
Le présent article résume et passe en revue les caractéristiques épidémiologiques de différentes maladies à prion humaines et les systèmes de surveillance les concernant dans différents pays.

Translated from English version into French by Suzanne Assenat, through

Эпидемиологические характеристики прионных заболеваний человека

Цао Чэнь, Сяо Пин Дун

Резюме

Прионные болезни человека – это группа трансмиссивных, прогрессирующих нейродегенеративных заболеваний с неизбежным летальным исходом, к которым относятся *Куру, болезнь Крейтцфельдта-Якоба (БКЯ), синдром Герстманна-Штреусслера-Шейнкера (СГШШ)* и *фатальная семейная инсомния (ФСИ).*
Прионные болезни человека могут иметь спорадический, наследственный и приобретённый характер. Ежегодно во всем мире регистрируются 1-2 случая заболевания на миллион человек. Эти заболевания привлекли внимание как научных кругов, так и общественности не только из-за своего загадочного возбудителя, но также из-за серьезной угрозы здоровью населения, после появления разновидности возбудителя, вызывающего *болезнь Крейтцфельдта-Якоба (БКЯ).*
До сих пор нет определённых терапевтических и профилактических средств для лечения *прионных заболеваний.* Поэтому активный контроль *прионных заболеваний* имеет решающее значение в борьбе с такого рода болезнями и при их профилактике. С 1993 года во многих странах и регионах были созданы системы мониторинга *болезни Крейтцфельдта-Якоба (БКЯ)*, а также проводились долгосрочные международные проекты по сотрудничеству в этой области.
В данной статье кратко излагаются и рассматриваются эпидемиологические характеристики *прионных заболеваний человека* и соответствующие системы активного наблюдения, применяемые в различных странах и регионах.

Translated from English version into Russian by Tatsiana Mankevich, through

Características epidemiológicas de las enfermedades priónicas humanas

Cao Chen, Xiao-Ping Dong

Resumen

Las enfermedades priónicas humanas son un grupo de enfermedades neurodegenerativas transmisibles, progresivas e inevitablemente mortales, que incluyen el kuru, la enfermedad de Creutzfeldt-Jakob (ECJ), el síndrome de Gerstmann-Sträussler-Scheinker y el insomnio familiar fatal. Las enfermedades priónicas humanas afectan aproximadamente a 1-2 personas por millón de habitantes a

nivel mundial por año. Pueden ser esporádicas, hereditarias o adquiridas. Estas enfermedades han atraído tanto la atención de científicos como del público no solo por lo misterioso de su agente patógeno, sino también por la considerable amenaza que representan para la salud pública desde el surgimiento de la variedad ECJ.

Todavía no existe disponibilidad de intervenciones terapéuticas y profilácticas específicas para las enfermedades priónicas. Por lo tanto, la vigilancia activa de las enfermedades priónicas humanas es vital para el control y prevención de la enfermedad. Desde el año 1993 se han establecido sistemas de vigilancia de la ECJ en varios países y regiones y se han llevado a cabo varios proyectos de cooperación multinacional a largo plazo.

En el presente artículo, se analizan y resumen las características epidemiológicas de varias enfermedades priónicas humanas y los sistemas de vigilancia activa que se utilizan para las mismas en distintos países y regiones.

Translated from English version into Spanish by Maria Alejandra Aguada, through

TRANSLATORS
WITHOUT BORDERS
